# Susceptibility of *Legionella gormanii* Membrane-Derived Phospholipids to the Peptide Action of Antimicrobial LL-37—Langmuir Monolayer Studies

**DOI:** 10.3390/molecules29071522

**Published:** 2024-03-28

**Authors:** Katarzyna Pastuszak, Małgorzata Jurak, Bożena Kowalczyk, Jacek Tarasiuk, Agnieszka Ewa Wiącek, Marta Palusińska-Szysz

**Affiliations:** 1 Department of Interfacial Phenomena, Institute of Chemical Sciences, Faculty of Chemistry, Maria Curie-Skłodowska University, Maria Curie-Skłodowska Sq. 3, 20-031 Lublin, Poland; katarzyna.pastuszak2@mail.umcs.pl (K.P.); agnieszka.wiacek@mail.umcs.pl (A.E.W.); 2 Department of Genetics and Microbiology, Institute of Biological Sciences, Faculty of Biology and Biotechnology, Maria Curie-Skłodowska University, Akademicka 19, 20-033 Lublin, Poland; bozena.kowalczyk@mail.umcs.pl (B.K.); jacek.tarasiuk@mail.umcs.pl (J.T.); marta.palusinska-szysz@mail.umcs.pl (M.P.-S.)

**Keywords:** *Legionella* bacteria, phospholipids, LL-37, Langmuir monolayers

## Abstract

LL-37 is the only member of the cathelicidin-type host defense peptide family in humans. It exhibits broad-spectrum bactericidal activity, which represents a distinctive advantage for future therapeutic targets. The presence of choline in the growth medium for bacteria changes the composition and physicochemical properties of their membranes, which affects LL-37’s activity as an antimicrobial agent. In this study, the effect of the LL-37 peptide on the phospholipid monolayers at the liquid–air interface imitating the membranes of *Legionella gormanii* bacteria was determined. The Langmuir monolayer technique was employed to prepare model membranes composed of individual classes of phospholipids—phosphatidylcholine (PC), phosphatidylethanolamine (PE), phosphatidylglycerol (PG), cardiolipin (CL)—isolated from *L. gormanii* bacteria supplemented or non-supplemented with exogenous choline. Compression isotherms were obtained for the monolayers with or without the addition of the peptide to the subphase. Then, penetration tests were carried out for the phospholipid monolayers compressed to a surface pressure of 30 mN/m, followed by the insertion of the peptide into the subphase. Changes in the mean molecular area were observed over time. Our findings demonstrate the diversified effect of LL-37 on the phospholipid monolayers, depending on the bacteria growth conditions. The substantial changes in membrane properties due to its interactions with LL-37 enable us to propose a feasible mechanism of peptide action at a molecular level. This can be associated with the stable incorporation of the peptide inside the monolayer or with the disruption of the membrane leading to the removal (desorption) of molecules into the subphase. Understanding the role of antimicrobial peptides is crucial for the design and development of new strategies and routes for combating resistance to conventional antibiotics.

## 1. Introduction

*Legionella* bacteria constitute the natural microflora of aquatic and soil environments. Once they penetrate artificial water distribution systems, these bacteria become a source of waterborne aerosols hazardous to human health and life. Upon entering the human body, *Legionella* spp. proliferate in alveolar macrophages, leading to the development of a type of pneumonia known as Legionnaires’ disease. *Legionella* bacteria are also a cause of flu-like infections called Pontiac fever. Legionnaires’ disease and Pontiac fever are collectively referred to as legionellosis. Due to advancements in diagnostics, along with shifts in demographics and climate, the incidence of legionellosis is steadily increasing [1]. Consequently, *Legionella* bacteria have emerged as the predominant waterborne pathogens in terms of their prevalence and the seriousness of the infections they cause. Most cases of legionellosis are caused by *L. pneumophila*, but other species of *Legionella* are also responsible for the disease. *L. gormanii* is one of the non-pneumophila clinically relevant species that cause infections mainly in individuals with weakened immunity, but infections have also been described in immunocompetent patients [2].

The intricate composition of bacterial membranes profoundly influences our understanding of pathogen behavior and their progression toward antibiotic resistance. The cell envelope of *L. gormanii* is typical of Gram-negative bacteria and consists of two distinct membranes, the inner membrane (IM) and the outer membrane (OM), separated by a periplasmic space. The outer leaflet of the OM is composed of lipopolysaccharides (LPSs), and the inner leaflet is made of phospholipids (PLs). The IM is built of phospholipids. The lipid profile of *L. gormanii* comprises phospholipids (phosphatidylcholine, PC; phosphatidylethanolamine, PE; phosphatidylglycerol, PG; cardiolipin, CL), neutral lipids (triglycerides, diglycerides), and sphingolipids (ceramides, hexosylceramides). The main classes of *L. gormanii* phospholipids are PE (50%) and PC (26%). The remaining phospholipids include CL (21%) and PG (3%) [3]. 

PC is the zwitterionic phospholipid that builds almost all eukaryotic cells. This component is considerably less prevalent in bacteria and is primarily found in species whose life cycle is associated with a eukaryotic host. PC deficiency in bacterial membranes significantly impacts the interaction of pathogens with the host cell and sensitivity to antibacterial peptides [4]. *L. pneumophila* synthesizes PC via two alternative pathways: the PE methylation pathway (PMT) and the PC synthase pathway (PCS) [5]. The PMT pathway is the successive three-fold N-methylation of PE catalyzed by phospholipid N-methyltransferases (Pmts). The PCS pathway encompasses the direct condensation of free choline with diacylglycerol-5’-diphosphocytidine (CDP-DAG), resulting in the formation of PC and cytidine monophosphate (CMP). The reaction is catalyzed by phosphatidylcholine synthase (Pcs), unique to bacteria [6]. The one-step synthesis pathway of PC, in which bacteria utilize extracellular choline, is the dominant biosynthesis pathway in *L. pneumophila* [7]. This pathway is more energetically favorable than the PMT pathway and allows bacteria to rapidly adjust their membrane physiology to adapt to changing environmental conditions. The PCS pathway may serve as an environmental sensor, as biosynthetic precursors present in lung tissue influence the virulence of *L. pneumophila* by incorporating PC into the cell envelope of these bacteria [7].

*L. gormanii* grown on a medium with choline synthesized 21% more PC, 12% less PE, and a 9% lower level of CL compared to the bacteria cultured on a medium without the addition of choline [8]. Each class of phospholipids exhibited its characteristic fatty acid (FA) pattern. In the PE class, nearly half of the acids consisted of branched-chain fatty acids, as follows: *i*16:0, *a*15:0, and *a*17:0 (where *i* indicates the methyl branch at the iso carbon atom and *a* means the location of the methyl branch at the anteiso carbon atom). This fraction was characterized by a high content of cyclopropane acid 17:0 (*cis*-9,10-methylenehexadecanoic acid) (17%) and hexadecanoic acid (18%). Cyclopropane acid 17:0 (19%) was dominant in the phosphatidylcholine fraction. This fraction contained both branched-chain (*i*16:0, *a*15:0) and straight-chain acids (16:0, 18:0). The PG class was characterized by a high amount of branched-chain fatty acids, namely *a*15:0 (16%), *i*16:0 (15%), and *a*17:0 (6%). Compared to other phospholipids, cardiolipin contained the highest amount of unsaturated acids (16:1Δ^9^, 18:1Δ^9^ (where Δ indicates the location of the unsaturated bond in the acid chain)) and long-chain fatty acids (from 20 to 24 carbon atoms) [9]. The complex composition of bacterial membranes, including their fatty acid profile, significantly impacts sensitivity to antibiotics and antimicrobial peptides.

Antimicrobial peptides (AMPs) represent a preserved class of peptides renowned for their broad-spectrum activity. They not only exhibit potent antimicrobial effects but also demonstrate immunomodulatory and anticancer properties [10]. With conventional antibiotics facing increasing bacterial resistance, AMPs are emerging as a promising alternative. Typically amphipathic, AMPs possess a high positive charge, enabling them to penetrate and disrupt the negatively charged membranes of bacterial cells. Naturally occurring peptides undergo structural modifications aimed at enhancing their pharmacokinetic properties, including absorption, half-life, metabolism, and bioavailability [11]. These alterations result in peptides that boast high selectivity and efficacy while ensuring safety and tolerability. Consequently, clinical research is underway for over 100 therapeutic peptides [12]. Several AMPs, such as gramicidin, daptomycin, colistin, vancomycin, oritavancin, dalbavancin, and telavancin, have been introduced for treating bacterial infections in humans [13].

Antimicrobial peptides naturally expressed in the cells of mammals, such as human cathelicidin LL-37, provide the first line of defense against infections. It is reported that LL-37 is capable of selectively disrupting the membrane components of microorganisms to present antibacterial effects [14]. Its activity can be modulated by modifying the structural composition and/or by increasing the net charge of bacterial membranes [15], which can result from variable conditions for growing bacteria. Even subtle changes in the phospholipid composition of bacterial cell membranes entail considerable differences in the susceptibility of cells to LL-37 and affect the mode of action applied by this peptide [16].

One of the key steps in peptide–membrane interactions is the initial contact of the peptide with the outer leaflet of the cell membrane. The Langmuir monolayer technique enables the construction of models of *Legionella* bacteria membranes, bringing us closer to understanding the physicochemical processes accompanying LL-37’s antimicrobial activity on the molecular level. In our previous study, we investigated the effect of the LL-37 peptide on the Langmuir monolayers constituted by the mixture of phospholipids isolated from *L. gormanii* cells cultured with or without exogenous choline (PL + choline and PL − choline, respectively) [17]. The research presented in this paper extends the foregoing ones to determine how the antimicrobial LL-37 peptide influences the Langmuir monolayers composed of individual phospholipid classes (PC, PE, PG, and CL) extracted from *L. gormanii* bacteria supplemented or not with exogenous choline and which class has the greatest impact on peptide action relative to the mixed membranes. For this purpose, the surface pressure–mean molecular area (π−A) compression isotherms were registered for the monolayers with and without peptide addition to the subphase at 20 °C and 37 °C. Based on these data, the compression modulus ($C_{S}^{-1})$ was calculated. Moreover, employing penetration tests, the effect of LL-37 on anionic (PG and CL) and zwitterionic (PC and PE) phospholipid monolayers was specified. The susceptibility of each PL class to the peptide was defined, allowing the characterization of the significance of each component (single class) for the mixed (multi-class, multi-component) model membrane interactions with LL-37. To the best of our knowledge, the use of individual classes of phospholipids extracted from *L. gormanii* cells for modeling bacterial membranes by the Langmuir technique has not been described in the literature so far. Monolayers of this type reflect the response of physiological membranes to the action of a peptide more faithfully than those formed of single compounds representing particular classes. The presented studies made it possible to propose the mechanism of action of the LL-37 peptide towards model bacterial membranes of *L. gormanii* cells.

## 2. Results

The studies presented in this paper were conducted in two ways. In the first one, π−A compression isotherms were obtained. They allowed for the determination of the interactions between PC, PE, PG, CL, and antimicrobial LL-37 peptide molecules at different stages of monolayer compression. In the second one, the adsorption isotherms were acquired by direct monitoring of the surface area changes over time while keeping a constant surface pressure of 30 mN/m in the monolayer. Such penetration tests led to an evaluation of the affinity of the peptide for phospholipids whose molecular packing density corresponded to the natural membrane of bacterial cells.

### 2.1. π−A Isotherms and Compression Modulus $C_{S}^{-1}-\pi$ Dependencies

**Figure 1:**
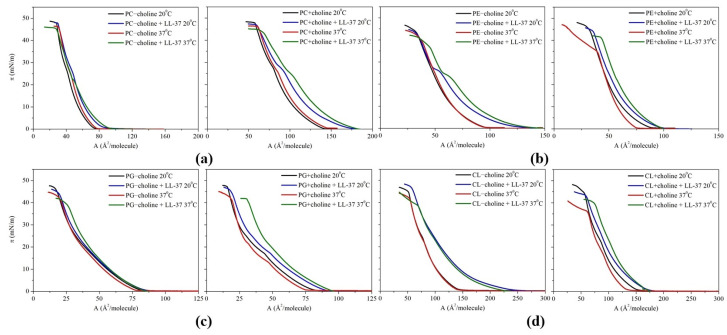
π−A isotherms obtained for the individual phospholipid classes (**a**) PC, (**b**) PE, (**c**) PG, and (**d**) CL isolated from *L. gormanii* bacteria cultured on a medium without (−choline) and with the addition of exogenous choline (+choline) at 20 °C and 37 °C, in the absence or presence of the LL-37 peptide.

The π−A isotherms, obtained for monolayers of PC, PE, PG, and CL classes isolated from *L. gormanii* bacteria cultured on a medium supplemented or not with exogenous choline in the absence or presence of the LL-37 peptide are presented in Figure 1. The experiments were conducted at two temperatures: 20 °C and 37 °C. 

Based on the attained dependencies, the lift-off area ($A_{0}$) and the collapse pressure ($\pi_{c}$) were defined. $A_{0}$ corresponds to the area at which the surface pressure reaches a detectable value of ca. 0.5 mN/m, while the collapse parameter describes the surface pressure value at which the two-dimensional monolayer is converted into the three-dimensional structures at the interface. To quantitatively depict the influence of the LL-37 peptide on each monolayer, the percentage differences between the aforesaid parameters characterizing the model membranes with and without the peptide were determined (Table 1). 

As can be seen in Figure 1 and Table 1, the presence of the LL-37 peptide in the subphase alters the behavior of the phospholipid molecules during the monolayer compression. The π−A isotherms registered in the presence of LL-37 are shifted towards larger mean molecular area values. Interestingly, the largest monolayer expansion is noted for CL − choline at both temperatures (60% at 20 °C and 52% at 37 °C) and the smallest for PG − choline (5% at 20 °C and 7% at 37 °C). Considering the collapse pressure, the decrease in this parameter resulting from the peptide addition is observed for most analyzed model membranes. This suggests that phospholipid monolayers formed in the presence of LL-37 are less stable than those without the peptide addition. Moreover, depending on the monolayer composition, some inflections in the course of the π−A isotherms or alterations in their slope registered in the presence of LL-37 in comparison to the pure phospholipid model membranes can be seen (Figure 1). Thus, the results confirm that the peptide accumulates at the interface and affects molecular organization during monolayer compression. This leads to local destabilization of the model membrane, even manifesting in the removal of some molecules into the subphase.

The further analysis of the physicochemical properties is based on the compression modulus parameter $C_{S}^{-1}$, which describes the degree of packing and ordering of the molecules in the monolayers. It is calculated directly from the π−A isotherms data, according to the formula presented below [18]:

$$C_{S}^{-1}=-A{\left(\frac{d\pi }{dA}\right)}_{T,p}$$

where p refers to the atmospheric pressure and T is the temperature.

**Figure 2:**
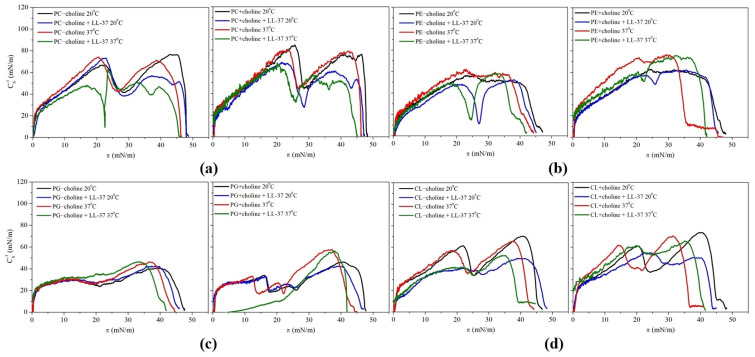
$C_{S}^{-1}-\pi$ dependencies obtained for the individual phospholipid classes (**a**) PC, (**b**) PE, (**c**) PG, and (**d**) CL isolated from *L. gormanii* bacteria cultured on a medium without (−choline) and with the addition of exogenous choline (+choline) at 20 °C and 37 °C, in the absence or presence of the LL-37 peptide.

The obtained results are presented as a function of the surface pressure in Figure 2, and the maximal $C_{S}^{-1}$ values are included in the Supplementary Materials as Table S1.

The maximum values of the compression modulus for all model membranes except PG − choline are in the range of 50–100 mN/m (Table S1), which is characteristic of monolayers in an intermediate state between liquid-expanded (LE) and liquid-condensed (LC) according to the Davies and Rideal criterion [18]. For PG − choline monolayers, the compression modulus does not exceed 50 mN/m, indicating the existence of only the LE phase, which most probably is the result of a large number of branched FA chains. 

The highest compression modulus values were obtained for PC monolayers (74–85 mN/m, Table S1). Based on these, it can be stated that the PC molecules form monolayers characterized by the greatest degree of packing and ordering, which can be assigned to the cylindrical shape of the molecules [19]. Without the peptide addition, a minimum on the $C_{S}^{-1}-\pi$ dependency is noted at π around 30 mN/m, indicating the second-order phase transition, presumably resulting from the changes in molecular tilting during the compression (Figure 2). The values assigned to the maxima are similar before and after the transition. Interestingly, the $C_{S}^{-1}-\pi$ curves obtained for the PC monolayers compressed with the addition of the LL-37 peptide also show the $C_{S}^{-1}$ decrease (a minimum) at around 30 mN/m. However, the values of the second maximum are much smaller than those of the first maximum, contrary to the PC monolayers without LL-37. Taking into account that the pure LL-37 peptide monolayer collapses at π around 24–30 mN/m [20,21], it can be assumed that the peptide-rich phase or the excess LL-37 is expulsed into the subphase when the monolayer is compressed to the mentioned surface pressure values. This manifests as the $C_{S}^{-1}$ decrease. The pushed-out peptide can be located in the adsorption layer underneath the headgroups, altering the fatty acid chain tilt [22]. This phenomenon most likely takes place not only in the case of PC monolayers but for PE − choline and PE + choline model membranes at both temperatures as well (Figure 2), in which, after the suggested peptide expulsion, the monolayers achieve approximately the same degree of packing as PE model membranes without LL-37 addition (Table S1). Contrarily, in anionic phospholipid monolayers, the compression modulus decrease is not unequivocally observed, which could indicate the desorption of molecules. A slight increase in the degree of packing or, at most, a 4 mN/m decrease occurs in PG monolayers (Table S1). Meanwhile, in CL model membranes, the $C_{S}^{-1}$ changes observed in pure phospholipid monolayers at π in a range of 20–30 mN/m do not exist after peptide addition. The most probable reason for such an influence of the peptide is that the PG and CL molecules are negatively charged, thus the electrostatic repulsion occurs in the monolayers, hindering the model membrane packing and molecule organization. Due to the fact that the LL-37 molecules are bearing a positive charge and they are interacting with the phospholipid molecules spread on the subphase, it is justified to assume that the peptide balances the repulsive interactions, resulting in membrane stabilization during the compression. Moreover, the cardiolipin monolayer analyzed after peptide addition shows, at most, a 23 mN/m decrease in the $C_{S}^{-1}$ maximal value (Table S1), which confirms the fluidizing effect of the LL-37 peptide on the molecular organization in the model membrane.

### 2.2. Penetration Studies (Adsorption Isotherms)

To define the mechanism of action of the LL-37 peptide on PC, PE, PG, and CL monolayers and to describe which of these components (classes) is most susceptible to the antimicrobial agent, penetration studies were carried out according to the constant surface pressure approach. Due to the fact that these analyses should mimic the LL-37 peptide influence on bacterial cells in human organisms as much as possible, the monolayers were compressed to 30 mN/m. This value is in agreement with the equilibrium lateral pressure of the biomembranes; the packing density of lipids is adequate for bilayers at this point [23,24]. Afterwards, the surface pressure was kept constant by the oscillating movement of barriers, resulting in alterations in the mean molecular area over time, depending on the behavior of the LL-37 molecules towards the phospholipid monolayer.

**Figure 3:**
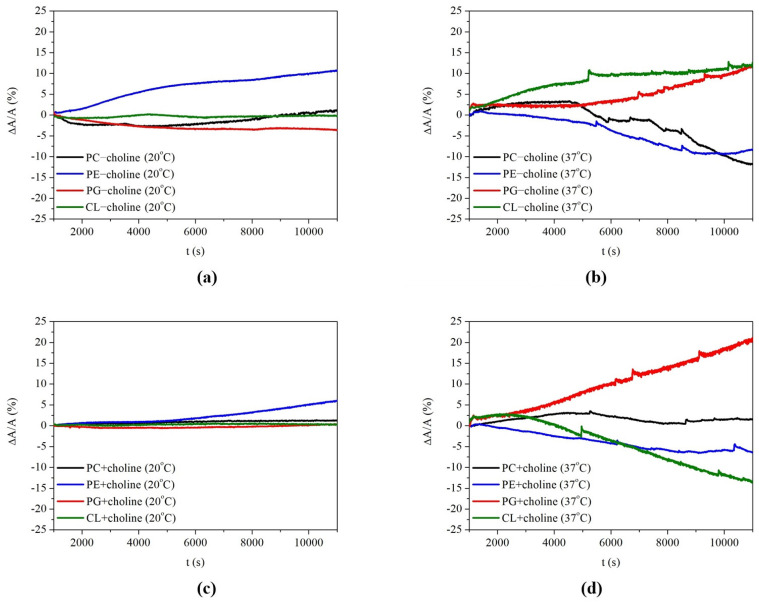
Relative area change ∆A/A as a function of time t, obtained for monolayers (**a**) “−choline” at 20 °C, (**b**) “−choline” at 37 °C, (**c**) “+choline” at 20 °C, and (**d**) “+choline” at 37 °C.

Figure 3 shows the relative area changes (∆A/A) over time for the single-class monolayers, caused by the addition of the LL-37 peptide to the subphase (adsorption isotherms). The ∆A/A parameter, expressed as a percentage, describes the difference in mean molecular areas registered in the presence and absence of the LL-37 peptide, referred to as the area per molecule in single-class model membranes [20].

As can be seen in Figure 3, both the presence of choline in the growth medium for bacteria and the temperature of the subphase have a significant influence on LL-37’s action towards single-class monolayers. The decrease in area per molecule caused by peptide addition can suggest the model membrane destabilization and desorption of some compounds from the monolayer into the subphase, while the increase in this parameter indicates peptide insertion into the compressed monolayers [20,21,25,26]. The relative area changes after 3 h of monolayer exposure to the peptide are presented in Table 2 for each phospholipid class. Based on these relationships, it can be noted that, depending on the PL class, different area changes in the monolayers associated with susceptibility to the peptide take place. 

In the “−choline” monolayers, at 20 °C, PE seems to be the most liable to peptide permeation, as it shows the biggest area increase, equal to 11% (Figure 3, Table 2). On the other hand, at 37 °C, the monolayers of both zwitterionic PC and PE phospholipids are characterized by a 12% and 10% decrease in ∆A/A, respectively, suggesting the desorption of molecules into the bulk phase. Meanwhile, for negatively charged PG and CL molecules, LL-37 can be incorporated into their monolayers, as revealed by the 14% and 12% increases in the ∆A/A parameter (Figure 3, Table 2). The phospholipid–peptide interactions are completely different in the “+choline” monolayers in comparison to the “−choline” ones. At 20 °C, none of the “+choline” monolayers is affected by LL-37 to a great extent. Interestingly, at 37 °C, the biggest deviations in the mean molecular area out of all the monolayers analyzed are noted, with a 22% increase in PG + choline and a substantial 14% decrease in CL + choline (Figure 3, Table 2). 

The above data clearly indicate that PE monolayers are mostly affected by LL-37 at 20 °C, while those of PG and CL are affected at 37 °C. These findings allow us to better understand the effect of the peptide on the behavior of the multi-class PL − choline and PL + choline membranes described in our previous paper [17]. This is explained in more detail in the Section 3 of this paper.

## 3. Discussion

Antimicrobial peptides, such as human cathelicidin, produced by various organisms ranging from the simplest to the human immune system, represent a new strategy for combating infections. In developing new antimicrobial peptide-based drugs, a key factor is understanding the complex interactions between these peptides and biological membranes using biophysical tools. Model membrane systems composed of phospholipids are employed to understand the mechanisms of AMP action at the molecular level, offering an alternative approach to studying membrane–peptide interactions with the advantage of easily controlling test conditions. Modifications in the structure of lipids and fatty acids affect the physicochemical properties of bacterial membranes and may determine their mechanism of interaction with antibacterial peptides. 

In this study, the Langmuir monolayer technique was used to mimic the bacterial membranes formed by phospholipid classes isolated from *L. gormanii* bacteria cultured on a medium with or without the addition of exogenous choline and to determine the effect of LL-37 on these membranes. Our studies indicate that LL-37 affects the conformation and orientation of the phospholipid molecules, significantly modifying the elasticity and stability of the monolayer and leading to its disintegration. This phenomenon is most noticeable for the zwitterionic PC and PE model membranes. For them, the initial incorporation is followed by the peptide expulsion from the monolayers after exceeding the collapse pressure of LL-37, i.e., 25–30 mN/m. This is revealed by inflections on the π−A isotherms and the minima of the compression modulus (Figure 1 and Figure 2), which can be indicative of the monolayers’ destabilization owing to the strong tendency of the components to phase segregation. Such an observation proves the repulsion interactions between the peptide and the phospholipids in the monolayer, resulting in the expulsion of the LL-37 molecules or the peptide-rich phase into the subphase. Contrarily, for the anionic PG and CL model membranes, the $C_{S}^{-1}$ minima do not occur (Figure 2); therefore, we can assume that the LL-37 molecules are situated in the monolayer, consequently affecting membrane elasticity. The presence of LL-37 in the negatively charged PG and CL monolayers promotes the expansion of the monolayer area, which, additionally, does not decrease with pressure (Figure 1, Table 1), implying stable peptide incorporation. 

**Figure 4:**
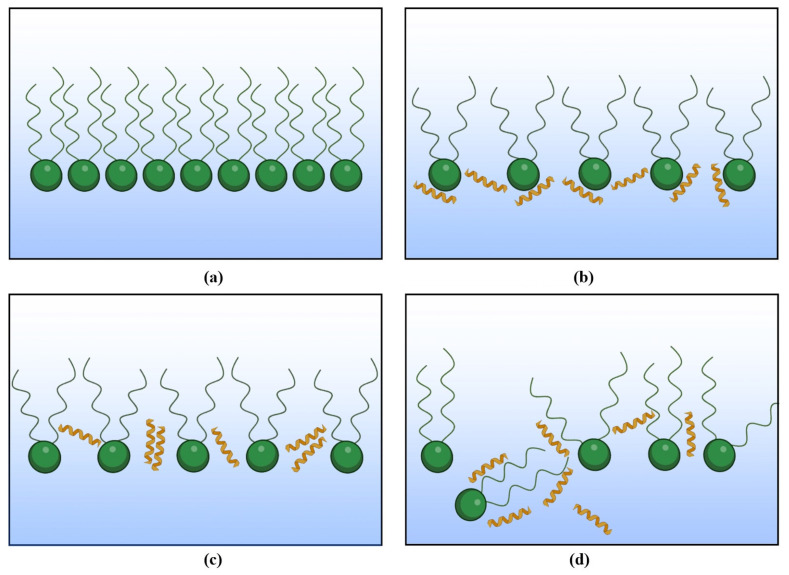
Proposed mechanisms of action of the LL-37 peptide on the phospholipid monolayers: (**a**) monolayer before peptide addition; (**b**) peptide located within the headgroup region of the model membrane; (**c**) peptide incorporation into the acyl chain region of the model membrane; (**d**) desorption of molecules from the model membrane into the subphase.

In addition to the π−A compression isotherm registration, monolayer penetration tests allow for evaluating the affinity of the peptide for phospholipids and proposing the probable action mechanisms of LL-37 (Figure 4a–d) by directly monitoring the surface area changes in the monolayer while keeping the surface pressure constant (adsorption isotherms, Figure 3). The phospholipid headgroups are the first region of the membrane faced with the peptide, and therefore, the charge, polarity, and size of these headgroups are decisive for the selectivity of LL-37. 

The PG and CL classes analyzed by the penetration study are found susceptible to peptide incorporation into the model membranes, especially at 37 °C, as pointed out by the greatest increase in the ∆A/A parameter (Figure 3, Table 2). This indicates that the positively charged LL-37 molecules are embedded in the anionic headgroup region of the monolayer due to the strong electrostatic interactions, as shown in Figure 4b. Similar observations and conclusions were previously made by other authors for the 1-palmitoyl-2-oleoyl-*sn*-glycero-3-phosphoglycerol (POPG) and 1,2-dipalmitoyl-*sn*-glycero-3-phosphoglycerol (DPPG) model membranes exposed to LL-37 [20,21,27]. This is in agreement with the above analysis of the π−A isotherms (Figure 1, Table 1) and the $C_{S}^{-1}-\pi$ dependencies (Figure 2, Table S1), which does not point to the peptide ejection into the subphase. Considering PG monolayers, the influence of LL-37 is more pronounced for the PG + choline model membrane, which is characterized by a greater degree of condensation at 30 mN/m (Figure 2, Table S1) and, thus, shorter distances between the molecules in comparison to PG − choline. A closer packing of anionic PG molecules can result in a higher concentration of negative charges at the interface. Consequently, more LL-37 molecules are strongly attracted towards PG + choline and bind with the headgroup region to a greater extent than with the PG − choline monolayer, leading to a significant area increase (Figure 3, Table 2). Contrarily, for CL + choline, a mean molecular area decrease (14%) is noted during the penetration tests, suggesting the desorption of molecules from the monolayer into the subphase (membrane destabilization; Figure 4d). As this model membrane is characterized by even greater packing and ordering than PG (Figure 2, Table S1), one can assume that electrostatic attraction causes significant disturbance and alterations in the monolayer structure and, in consequence, the fatty acid chain orientation at the interface, enabling stronger Lifshitz–van der Waals interactions between CL and LL-37. A similar dependency was previously observed for PL − choline and more densely packed PL + choline model membranes exposed to LL-37 [17]. 

Zwitterionic compounds (PC and PE) are affected by the LL-37 peptide in a completely different way from anionic ones. The analyses conducted by other researchers usually suggest little to no effect of the LL-37 peptide on one-component 1-palmitoyl-2-oleoyl-*sn*-glycero-3-phosphocholine (POPC) or 1,2-dipalmitoyl-*sn*-glycero-3-phosphocholine (DPPC) monolayers [20,27]. This is in agreement with the significantly smaller susceptibility of the zwitterionic monolayers in comparison to the anionic ones analyzed in our studies. However, the PE membrane destabilization described above suggests lower monolayer resistance to LL-37 than was previously determined, e.g., by Sevcsik [21]. The main reason can be the diversity of fatty acid chains in *L. gormanii* PE monolayers, which determine a different packing of molecules than in PE model membranes containing one type of FA chain. The peptide embedding observed in our studies is more pronounced for less densely packed PE − choline (Figure 2, Table S1) in comparison to PE + choline. The expansion of the PE − choline monolayers can be associated with a greater content of cyclopropane acid *c*17:0 [9]. The presence of a cyclopropane ring structure increases the spacing between hydrocarbon chains by reducing their ability to pack tightly due to the weakening of the Lifshitz–van der Waals forces that stabilize chain–chain interactions. Moreover, electrically neutral PE can form hydrogen bonds with amino acids in the LL-37 structure due to the presence of $-NH_{3}^{+}$ and $-OPO_{3}^{-}-$ [28], promoting peptide embedding inside the monolayer. The longer distances between molecules limit the creation of PE-PE hydrogen bonds in favor of LL-37. At 37 °C, the H bonds are constantly formed and broken as a result of the thermal motions of molecules and greater kinetic energy [29,30]. Therefore, the peptide molecules can interact with FA chains to a greater extent (Figure 4c), which results in the desorption of some molecules from the monolayer (Figure 4d), as revealed by the decrease in molecular area (Figure 3, Table 2). 

Only a slight effect in comparison to the other classes evoked by LL-37 in PC model membranes (Figure 1, Table 1) correlates with the observations made on the basis of the penetration study results. In general, the PC monolayer is the most resistant to the action of LL-37 out of the individual phospholipid classes analyzed (Figure 3, Table 2). This phenomenon is probably related to the PC headgroup structure and its orientation at the interface. The peptide interactions with the polar headgroups are hindered due to the repulsion occurring between the cationic LL-37 and the positively charged ${-N}^{+}(CH_{3}{)}_{3}$ groups in PC, which are oriented towards the subphase, shielding the $-OPO_{3}^{-}-$ groups [31]. Nevertheless, these forces do not appear to be dominant, and the peptide molecules can penetrate towards the hydrophobic region of the membrane (Figure 4c) under certain experimental conditions. Owing to the mutual interactions between the peptide and the FA chains, the desorption of molecules from the monolayer can occur (Figure 4d), as it was found for the PC − choline monolayer at 37 °C (Figure 3, Table 2). The PC − choline model membrane contains more branched saturated fatty acid chains (*a*15:0 and *i*16:0) in comparison to PC + choline, which can promote looser packing. Therefore, the fatty acid chains are more available for the peptide, and stronger Lifshitz–van der Waals interactions between FAs and LL-37 can take place. This fact, in addition to the thermal motions of molecules at higher temperatures altering the chains’ accessibility to the peptide, provokes model membrane destabilization.

As described previously in [17], the PL − choline and PL + choline model membranes show various behaviors at the interface in the presence of the LL-37 peptide, which is strongly dependent on phospholipid composition and measurement temperature. The analysis of LL-37’s influence on single-class monolayers allows one to define which class mainly determines the susceptibility of multi-class model membranes to the peptide. The PE class constitutes 50% of the PL − choline mixture and 38% of PL + choline [9], and the results presented in this paper show that PE at 20 °C is the most susceptible to LL-37 incorporation out of all the analyzed classes. In consequence, it can be assumed that PE’s behavior in the presence of the peptide is decisive for the peptide–mixture interactions and defines the 25% and 18% mean molecular area increase registered for PL − choline and PL + choline, respectively [17]. However, at 37 °C, the mutual interactions of PE with LL-37 do not seem to be crucial, as in these conditions, the peptide’s influence is dominant on PG and CL single-class monolayers, and in the case of the “−choline” membranes, the greatest LL-37 incorporation is noted (Figure 3, Table 2). Moreover, the higher temperature enhances the repulsive interactions between phospholipids in multi-component monolayers, possibly provoking stronger electrostatic interactions between anionic PG or CL and the peptide. Therefore, the anionic phospholipids seem to be the main component defining the 56% area augmentation in the quaternary model membrane [17]. CL + choline at increased temperature is the least resistant to the antimicrobial agent action, showing a 14% relative area decrease (Figure 3, Table 2), which is responsible for PL + choline’s critical destabilization and a 70% area decrease [17].

## 4. Materials and Methods

The phospholipids (phosphatidylcholine, PC; phosphatidylethanolamine, PE; phosphatidylglycerol, PG; cardiolipin, CL) were isolated from *Legionella gormanii* bacteria cultured without (−choline) or with exogenous choline (+choline). Details of this procedure regarding bacterial strains and culture conditions, extraction of lipids, and separation into individual classes are presented in Supplementary Materials. The phospholipids were dissolved in chloroform (Avantor Performance Materials Poland S.A., Gliwice, Poland; purity > 99.9%) and methanol (ROMIL Chemicals Ltd., Cambridge, UK; purity > 99.9%) at a volume ratio of 4/1, and 1 mg/mL solutions were obtained. The Langmuir–Blodgett (KSV 2000 Standard, KSV Instruments, Helsinki, Finland) and Langmuir (KSV Nima, Biolin Scientific, Stockholm, Sweden) troughs equipped with symmetric barriers and a Wilhelmy plate for surface tension determination (accuracy of 0.1 mN/m) were used to acquire the surface pressure–mean molecular area (π−A) isotherms. A 0.01% acetic acid solution, obtained by diluting the concentrated acetic acid (Avantor Performance Materials Poland S.A., Gliwice, Poland, 99.7%) with Milli-Q water (resistivity 18.2 MΩcm), was utilized as a subphase for all experiments. An external water circulating system (Lauda, Schwechat, Austria) was used to keep a constant temperature (20 °C or 37 °C; ±0.1 °C) during the measurements. The solutions were applied to the subphase surface using a Hamilton microsyringe. To ensure that the volatile solvents were fully evaporated, the trough was left for 10 min. Then, the monolayer was compressed with the symmetric barriers at a rate of 10 mm/min until the film collapsed. Each experiment was repeated 2–3 times, giving a mean error of 2 ${Å}^{2}$/molecule. Compression measurements with the addition of the LL-37 peptide (LL-37 (human) trifluoroacetate salt, Merck, Darmstadt, Germany; purity ≥ 95%) were conducted by dropping the peptide solution (1 mg/mL) onto the subphase surface, obtaining a concentration of 0.08 µg/mL in the bulk phase. The cathelicidin solution was left on the subphase surface for 2 h, and then the phospholipid solution was applied. The peptide-to-phospholipid molar and mass ratios were equal to 1:73 and 1:13, respectively. The measurements of π−A isotherms were then carried out as described above. 

The tests of the monolayer penetration by the LL-37 peptide were conducted in the following manner: The phospholipids dissolved in chloroform/methanol mixture were dropped onto the subphase surface and compressed to a surface pressure of 30 mN/m. After reaching this point, the surface pressure was maintained through oscillating barrier movements with a speed equal to 5 mm/min. Then, after 15 min of monolayer equilibration, the LL-37 peptide was injected into the subphase underneath the spread monolayer. The changes in mean molecular area vs. time were registered for about 3 h. As a control, measurements without the peptide were conducted analogously for the same period of time.

## 5. Conclusions

In this study, Langmuir monolayers composed of individual phospholipid classes (PC, PE, PG, CL) isolated from *L. gormanii* bacteria cultured on a medium with or without the addition of exogenous choline were analyzed to determine their physicochemical properties, susceptibility to the LL-37 peptide, and the influence of particular components on the multi-class monolayer behavior in the presence of the antimicrobial peptide.

The obtained results demonstrate that all the analyzed model membranes are in the LE or intermediate LE-LC state and undergo expansion in the presence of LL-37 in the subphase. The type of interactions and antimicrobial mechanisms of action of LL-37 are strongly dependent on the subphase temperature, with greater peptide influence observed at 37 °C, and the phospholipid structure. For zwitterionic PC and PE model membranes, peptide expulsion into the subphase can occur, while in anionic PG and CL monolayers, due to the strong electrostatic interactions with the peptide, LL-37 molecules are able to stably incorporate into the membrane. The penetration studies allowed us to propose two possible mechanisms of action, namely adsorption of the LL-37 molecules in the headgroup region of the monolayer or insertion into the fatty acid chain region. The first phenomenon is more pronounced in the case of anionic PG and CL model membranes, and the second one seems to be dominant in PC and PE monolayers. However, it is important to note that even if one mechanism is decisive under certain conditions, the other types of interactions cannot be ruled out, as the effect of the LL-37 peptide is not unambiguous. The monolayers characterized by a greater degree of packing and ordering are more susceptible to the peptide, suggesting preferential interactions of the LL-37 peptide with less elastic model membranes. This phenomenon is observed in multi-class PL monolayers, as PL + choline exhibits greater membrane destabilization. Contrarily, for PL − choline, peptide influence is less pronounced. This monolayer is characterized by a lower degree of order. Hence, LL-37 molecules can interact with the phospholipids without causing drastic alterations in the membrane. The composition of a multi-class model membrane is crucial for its molecular organization and behavior in the presence of the peptide. PE molecules seem to define the susceptibility of the mixed membranes to the peptide observed at 20 °C, while anionic PG and CL are most likely responsible for membrane destabilization at 37 °C.

A more profound insight into the interactions between LL-37 and bacterial membranes obtained in this paper can be helpful for the further design and development of peptide-based therapeutics that show greater selectivity against Gram-negative bacteria.

## Figures and Tables

**Table 1:** Changes in the lift-off area ($∆A_{0}$) and the collapse surface pressure ($∆\pi_{c}$) caused by the LL-37 peptide addition obtained for the “−choline” and “+choline” (a) PC, (b) PE, (c) PG, and (d) CL monolayers at 20 °C and 37 °C.

**(a)**	$∆A_{0}$ ** [%]**	$∆\pi_{c}$ ** [%]**	**(b)**	$∆A_{0}$ ** [%]**	$∆\pi_{c}$ ** [%]**
PC − choline 20 °C	22.8	0.2	PE − choline 20 °C	35.6	−0.2
PC − choline 37 °C	20.3	−1.9	PE − choline 37 °C	43.4	−1.0
PC + choline 20 °C	23.5	−2.7	PE + choline 20 °C	13.6	−4.7
PC + choline 37 °C	23.1	−3.0	PE + choline 37 °C	30.3	−1.9
**(c)**	$∆A_{0}$ ** [%]**	$∆\pi_{c}$ ** [%]**	**(d)**	$∆A_{0}$ ** [%]**	$∆\pi_{c}$ ** [%]**
PG − choline 20 °C	5.0	−3.7	CL − choline 20 °C	60.1	2.9
PG − choline 37 °C	7.3	−6.2	CL − choline 37 °C	52.1	−9.2
PG + choline 20 °C	9.2	−3.6	CL + choline 20 °C	10.0	−6.9
PG + choline 37 °C	17.4	−0.2	CL + choline 37 °C	19.9	9.9

**Table 2:** Relative area changes ∆A/A obtained for “−choline” and “+choline” (a) PC, (b) PE, (c) PG, and (d) CL monolayers at 20 °C and 37 °C, about 3 h after LL-37 injection.

**(a)**	$∆A/A_{3h}$ ** [%]**	**(b)**	$∆A/A_{3h}$ ** [%]**
PC − choline 20 °C	1.4	PE − choline 20 °C	10.9
PC − choline 37 °C	−12.2	PE − choline 37 °C	−9.5
PC + choline 20 °C	1.4	PE + choline 20 °C	6.2
PC + choline 37 °C	1.9	PE + choline 37 °C	−6.8
**(c)**	$∆A/A_{3h}$ ** [%]**	**(d)**	$∆A/A_{3h}$ ** [%]**
PG − choline 20 °C	−3.8	CL − choline 20 °C	−0.2
PG − choline 37 °C	14.0	CL − choline 37 °C	12.2
PG + choline 20 °C	0.4	CL + choline 20 °C	0.4
PG + choline 37 °C	22.4	CL + choline 37 °C	−14.0

## Data Availability

Data are contained within the article and Supplementary Materials.

## Supplementary Materials

The following supporting information can be downloaded at: https://www.mdpi.com/article/10.3390/molecules29071522/s1, Table S1: The maximal ($C_{S,max}^{-1}$) and minimal ($C_{S,min}^{-1}$) compression modulus values obtained for the “−choline” and “+choline” (a) PC (b) PE (c) PG and (d) CL monolayers at 20°C and 37°C, in the absence or presence of the LL-37 peptide. The $C_{S}^{-1}$ and $\pi_{C_{S}^{-1}}$ values are given in mN/m; Materials and methods: Bacterial strain and culture conditions; Extraction of lipids; Purification and separation of phospholipids by TLC. References [32,33] are cited in the Supplementary Materials.
