# Supplementary material for: Susceptibility of Legionella gormanii Membrane-Derived Phospholipids to the Peptide Action of Antimicrobial LL-37—Langmuir Monolayer Studies
Source: Molecules. 2024 Mar 28;29(7):1522. doi: 10.3390/molecules29071522 (PMC11013288; doi:10.3390/molecules29071522)
Supplement: Supplementary file 1 [file molecules-29-01522-s001.zip › molecules-2897981-supplementary.pdf]

molecules

MDPI

Supplementary Materials

Susceptibility of *Legionella gormanii* Membrane-Derived Phospholipids to the Peptide Action of Antimicrobial LL-37—Langmuir Monolayer Studies

Katarzyna Pastuszak [1], Małgorzata Jurak [1,*], Bożena Kowalczyk [2], Jacek Tarasiuk [2], Agnieszka Ewa Wiącek [1], Marta Palusińska-Szysz [2]

[1] Department of Interfacial Phenomena, Institute of Chemical Sciences, Faculty of Chemistry, Maria Curie-Skłodowska University, Maria Curie-Skłodowska Sq. 3, 20-031 Lublin, Poland; katarzyna.pastuszak2@mail.umcs.pl (K.P.); agnieszka.wiacek@mail.umcs.pl (A.E.W.)

[2] Department of Genetics and Microbiology, Institute of Biological Sciences, Faculty of Biology and Biotechnology, Maria Curie-Skłodowska University, Akademicka 19, 20-033 Lublin, Poland; bozena.kowalczyk@mail.umcs.pl (B.K.); jacek.tarasiuk@mail.umcs.pl (J.T.); marta.palusinska-szysz@mail.umcs.pl (M.P.-S.)

* Correspondence: malgorzata.jurak@mail.umcs.pl

Supplementary Materials

Citation: To be added by editorial staff during production.

Academic Editor: Firstname Lastname

Received: date
Revised: date
Accepted: date
Published: date

Copyright: © 2024 by the authors. Submitted for possible open access publication under the terms and conditions of the Creative Commons Attribution (CC BY) license (https://creativecommons.org/licenses/by/4.0/).

Table S1. The maximal ($C_{S,max}^{-1}$) and minimal ($C_{S,min}^{-1}$) compression modulus values obtained for the "−choline" and "+choline" (a) PC (b) PE (c) PG and (d) CL monolayers at 20°C and 37°C, in the absence or presence of the LL-37 peptide. The C_S^{-1} and $\pi_{C_S^{-1}}$ values are given in mN/m.

**(a)**	$C_{S,max1}^{-1}$	$\pi_{C_{S,max1}^{-1}}$	$C_{S,min}^{-1}$	$\pi_{C_{S,min}^{-1}}$	$C_{S,max2}^{-1}$	$\pi_{C_{S,max2}^{-1}}$
PC − choline 20 °C	67.1	22.5	41.2	28.7	76.6	44.3
+ LL-37	73.2	22.8	38.3	28.7	56.9	37.2
PC − choline 37 °C	74.4	20.5	42.9	26.7	71.2	38.7
+ LL-37	62.6	23.7	43.7	28.5	50.5	32.6
PC + choline 20 °C	84.4	25.5	44.0	29.6	76.3	46.3
+ LL-37	68.9	21.6	27.2	28.6	60.7	38.3
PC + choline 37 °C	80.7	23.2	43.4	26.8	79.2	42.3
+ LL-37	68.1	20.1	32.2	25.8	57.1	31.5
**(b)**	$C_{S,max1}^{-1}$	$\pi_{C_{S,max1}^{-1}}$	$C_{S,min}^{-1}$	$\pi_{C_{S,min}^{-1}}$	$C_{S,max2}^{-1}$	$\pi_{C_{S,max2}^{-1}}$
PE − choline 20 °C	57.1	25.6	-	-	-	-
+ LL-37	48.0	21.7	11.5	27.1	52.4	38.0
PE − choline 37 °C	61.9	23.0	-	-	-	-
+ LL-37	48.7	18.4	15.1	24.6	57.9	33.0
PE + choline 20 °C	62.9	24.5	-	-	-	-
+ LL-37	57.1	22.6	49.2	25.9	62.3	35.7
PE + choline 37 °C	76.2	29.6	-	-	-	-
+ LL-37	60.7	20.5	51.4	22.4	75.7	32.5
**(c)**	$C_{S,max1}^{-1}$	$\pi_{C_{S,max1}^{-1}}$	$C_{S,min}^{-1}$	$\pi_{C_{S,min}^{-1}}$	$C_{S,max2}^{-1}$	$\pi_{C_{S,max2}^{-1}}$
PG − choline 20 °C	30.2	12.9	23.6	21.3	40.6	39.6
+ LL-37	30.1	14.0	26.7	20.4	42.4	37.4
PG − choline 37 °C	30.7	12.7	25.6	20.3	46.6	36.8
+ LL-37	46.3	33.2	-	-	-	-
PG + choline 20 °C	34.3	16.2	20.2	26.1	46.4	41.0
+ LL-37	32.8	16.4	22.6	25.8	42.4	39.4
PG + choline 37 °C	33.0	12.3	17.2	22.1	57.7	37.1
+ LL-37	56.4	38.2	-	-	-	-
**(d)**	$C_{S,max1}^{-1}$	$\pi_{C_{S,max1}^{-1}}$	$C_{S,min}^{-1}$	$\pi_{C_{S,min}^{-1}}$	$C_{S,max2}^{-1}$	$\pi_{C_{S,max2}^{-1}}$
CL − choline 20 °C	61.1	22.0	34.0	25.5	70.5	40.7
+ LL-37	40.4	22.1	34.4	28.5	49.7	40.9
CL − choline 37 °C	56.8	18.0	35.6	23.3	65.3	37.4
+ LL-37	41.6	21.4	34.1	25.4	52.4	35.1
CL + choline 20 °C	60.7	20.5	36.7	24.4	73.5	40.1
+ LL-37	54.8	24.3	43.7	31.1	50.2	39.1
CL + choline 37 °C	61.8	14.2	38.4	21.7	70.5	31.4
+ LL-37	61.5	19.9	52.4	25.6	65.5	35.1

Materials and methods

Bacterial strain and culture conditions

L. gormanii (ATCC 33297) was grown either on buffered charcoal yeast extract (BCYE) agar plates (Oxoid, Basingstoke, UK) or on this medium supplemented with 100 µg/mL choline chloride (Sigma-Aldrich, St. Louis, MO, USA) at 37 °C and 5% CO_2 in

a humid environment for 3 days. The biomass was collected by centrifugation at 8000 rpm for 20 minutes, followed by two washes with 0.5 M NaCl, one wash with water purified by the Milli-Q system, and subsequent freeze-drying.

Extraction of lipids

Lipids of *L. gormanii* cultured on BCYE medium with or without the addition of exogenous choline were isolated according to the Bligh and Dyer method [32] using a chloroform/methanol mixture (1/2, v/v). Suspended bacterial mass was vigorously mixed for 4 hours and centrifuged for 30 min, 6000×g (where ×g means times gravity), 4 °C. The pellet was resuspended in a new portion of chloroform/methanol (1/2, v/v). Lipid extraction was continued for 3 h and centrifuged for 30 min, 6000×g, 4 °C. After pooling the organic phases, chloroform and water were added to the final proportion of methanol/chloroform/water (2/2/1.8 ,v/v/v) and centrifuged for 30 min, 6000×g, 4 °C. The lower organic phase was collected and then concentrated on a rotary evaporator.

Purification and separation of phospholipids by TLC

The one-dimensional thin-layer chromatography (TLC) was performed in order to remove the pigments from extracted phospholipids, especially the exhibiting blue-white autofluorescence under the long-wavelength UV light legioliulin [33]. The 10 cm x 10 cm silica gel 60 F254 plates (Merck, Darmstadt, Germany) were first washed with chloroform/methanol (1/1, v/v) solvents at room temperature to remove contaminants. After the plates dried, about 2 mg of phospholipids dissolved in chloroform/methanol mixture (4/1, v/v) were applied on the start line and developed in chloroform/methanol/acetic acid (98/2/1, v/v/v) system preceded by 20 minutes of conditioning in solvent vapours. The pigments were visualized under the long-wavelength UV (Transiluminator UV-953) and the phospholipid bands were detected utilizing iodine vapour, scraped off the plate, and transferred to the screw-capped tubes. The 3 mL of chloroform/methanol (1/1, v/v) mixture was used to separate the silica from lipids. Purified phospholipids were then dried under nitrogen gas, weighted, and stored at −20 ℃ before separation into individual classes.

To conduct the separation into classes, the PLs were dissolved in chloroform/methanol (4/1, v/v), applied on a silica plate (about 2 mg), and developed using chloroform/methanol/glacial acetic acid (13/5/2, v/v/v) mixture. To identify the classes' retention order on the TLC plate, the PC, PE, PG, and CL standard solutions were also developed in the same solvent system. The phospholipids were visualized in iodine vapour and then scrapped, separated from the silica, evaporated to dryness, and weighted as before.
